# Effect of Rice Bran Protein on the Foaming Properties and Foaming Characteristics of Rice Bran Protein–Sodium Caseinate and Rice Bran Protein Nanoparticles–Sodium Caseinate

**DOI:** 10.3390/foods13152328

**Published:** 2024-07-24

**Authors:** Yanpeng Zhang, Delong Li, Yunchun Diao, Wei Xu, Guozhen Wang, Zhixiong Hu, Chun Hu

**Affiliations:** 1 Key Laboratory for Deep Processing of Major Grain and Oil, Ministry of Education, Wuhan Polytechnic University, Wuhan 430023, China; 2 College of Food Science and Engineering, Wuhan Polytechnic University, Wuhan 430023, China; ldl7128@163.com (D.L.); adiao6613@163.com (Y.D.); xuwei1216@163.com (W.X.); gzwang1988@163.com (G.W.); e_huzhixiong@126.com (Z.H.); huchun202020@163.com (C.H.)

**Keywords:** rice bran protein nanoparticles, foaming properties, physicochemical characteristics, interfacial rheological properties

## Abstract

Rice bran, a byproduct of rice milling, comprises 12–14% protein. The foaming properties and associated mechanisms of the composite rice bran protein system were not well studied. In this study, a composite protein system composed of rice bran protein (RBP)–sodium caseinate (NaCas) and rice bran protein nanoparticles (RBPNs)–sodium caseinate (NaCas) was investigated. The results showed that the synergistic effect of RBP and NaCas increased the foaming stability of the composite solution up to 83.77 ± 2.75%. Moreover, the foaming capacity and foaming stability of the RBPNs-NaCas composite solution were up to 177.50 ± 3.53% and 80.28 ± 0.39%, respectively. The physicochemical properties results revealed that the particle size volume peaks of RBP-NaCas and RBPNs-NaCas were mainly concentrated at 55.7 nm and 197.1 nm, and RBPNs-NaCas showed a wider single peak particle size distribution. The ζ-potential values of RBP-NaCas and RBPNs-NaCas were changed to −35.5 ± 0.07 mV and −27.2 ± 0.28 mV after complexation. The apparent viscosity and consistency factor of RBP-NaCas decreased by 31.1% compared to RBP, while RBPNs-NaCas displayed similar parameters to the single proteins. The interfacial rheological test showed that RBP and RBPNs can significantly improve the interfacial properties of NaCas by enhancing the interfacial interaction and the interfacial viscoelastic modulus of composite proteins, which is conducive to the stability of the foam system. The outcome of the study provided a theoretical basis for RBP and RBPNs to partially replace NaCas in the processing of foamed food.

## 1. Introduction

Foam is the multiphase dispersed colloidal system providing a charming visual sensation and smooth texture for the food system [1], which is essential to the sensory properties of aerated foods such as whipped cream, meringue, mousse, cake, etc. [2,3]. However, foams can coagulate, disintegrate, and coalesce within a short period due to the mechanisms of disproportionation, liquid drainage, and film rupture, making it challenging to mass manufacture and maintain such a thermodynamically unstable system [4]. The enhancement of foaming stability by constructing a highly viscoelastic interfacial structure through small-molecule surfactants with high interfacial mobility and macromolecular polymers to prevent foams in the dispersed phase from coalescing and coarsening is often used. With the development of the modern food processing industry, the application of natural and green surfactants in food processing is also getting more and more attention [5]. Therefore, how to build a nutritious and healthy food foam system has attracted the attention of many food scientists.

Sodium caseinate (NaCas), derived from bovine milk protein, has a flexible structure and relatively high solubility, allowing it to adsorb rapidly at the air–water interface; therefore, it is extensively used as a foaming agent in the food industry [6]. However, due to the tendency to form adsorption layers similar to a thin three-dimensional gel structure, the foaming stability of NaCas is far inferior compared with whey protein and egg white protein [7], which limits its further application in foamed foods. Compared to the flexible structure of NaCas, plant proteins exhibit a rigid, compact conformation, which facilitates the formation of a highly viscoelastic interfacial film and protein aggregation in solution to stabilize the foam system [8,9]. The relative finding showed that there was a synergistic effect between 7s soy globulin and β-lactoglobulin at pH 7.0 after mixing in an equal ratio, and the protein mixture produced denser, smaller bubbles with higher stability [10]. Moreover, it was proven that the co-precipitation of the whey protein isolate and pea protein complex enhanced the interfacial viscoelasticity and foaming stability [11]. The findings of these studies indicate that a strategy of compounding two kinds of proteins in different structures as a foaming agent is beneficial to improving the single protein foam system. Additionally, developing plant–animal composite proteins can also balance the amino acid composition and improve nutritional values.

Rice bran is a byproduct of rice milling, and it is composed of an abundant amount of protein [12]. Rice bran protein (RBP) is recognized as one of the optimal proteins with a well-balanced amino acid profile, high bioavailability, and hypoallergenicity among the known whole grain or legume proteins [13,14]. Recently, it has been discovered that protein nanoparticles can form a viscoelastic film around bubbles at the air–water interface through molecular adsorption, structural unfolding, and molecular rearrangement [15,16]. For example, Wang et al. found that the caseinate–soy protein nanoparticle (Cas-SPNP) complex prepared at a ratio of 7:3 at pH 12.0 showed better interface characteristics and superior foaming properties [16]. This finding highlighted that the protein nanoparticles are promising new surface-active ingredients for stabilizing foamed food made of caseinate-based composite proteins. The same research group in this study found that RBP has good foaming properties, and the foaming properties of RBP nanoparticles (RBPNs) prepared by acid–heat treatment can be significantly improved [17,18]. However, the relevant research about the effect of RBP and RBPN complexes with NaCas on the formation of viscoelastic interface films and foaming properties is still extremely limited, especially since the role of protein nanoparticles in stabilizing the foam of the NaCas-based composite protein system has not been well studied. Therefore, it is essential to investigate the effect of intermolecular interactions on interfacial adsorption and viscoelastic membrane structure in composite protein systems so as to explore the feasibility of using RBP and RBPNs as a new source of partial replacement of animal protein in the industrial processing of foamed foods.

In this study, the RBP-NaCas and RBPNs-NaCas composite protein systems were prepared by mixing in equal proportion, and the relevant changes in the foaming characteristics of the composite protein system at pH 7.0 as a representative condition of food systems were investigated. This study provides a distinctive perspective on how composite protein systems affect the body phase, interface behavior, and foaming properties, which reveals the possibility of RBP as a natural foaming agent and provides guidance for the partial replacement of NaCas with RBP and RBPNs in foamed food processing and theoretical research.

## 2. Materials and Methods

### 2.1. Materials

The fresh rice bran (RB) was kindly provided by the local rice processing company (E Zhou, China) and stored at a constant temperature of −20 °C. The sodium caseinate (NaCas) was purchased from Sigma (AR, Shanghai, China). Other chemicals and reagents used in this study were assured to be analytically pure and commercially purchased from Sinopharm (Beijing, China) Co., LLC. All solutions and dispersions were prepared using deionized water.

### 2.2. Preparation of Rice Bran Protein and Rice Bran Protein Nanoparticles

The extraction of RBP and the preparation of RBPNs were performed according to the method of Zhang et al. [17]. The rice bran was crushed and sieved through an 80-mesh sieve, and the oil was extracted 5 times with n-hexane at 55 °C in a ratio of 1:5 (*w*/*v*). The defatted rice bran powder was mixed with distilled water at a ratio of 1:10 (*w*/*v*) at pH 9.0. After 2 h of extraction, the solution was centrifuged at 10,000 rpm for 15 min to collect the supernatant. The pH value of the supernatant was then adjusted to 4.5 to precipitate protein by centrifugation at 8000 rpm for 15 min. The protein precipitate was washed 3 times with deionized water and then freeze-dried. The extracted RBP (80.5% protein content) was stored in a refrigerator at 4 °C for spare, and the protein content of the RBP samples was determined by the Micro-Kjeldahl method [18,19].

The RBP powder was dissolved in deionized water to form a solution with a protein concentration of 1.5% (*w*/*v*) and hydrated overnight at 4 °C. Subsequently, the pH of the RBP solution was adjusted to 2.0 using 1.0 mol/L HCl and stirred for 2 h, followed by centrifugation at 5000 rpm for 10 min at 25 °C to remove the insoluble components. The RBP suspension was heated at 90 °C for 24 h and then cooled in an ice bath to terminate the reaction. The RBPNs sample was prepared after a freeze-drying treatment and stored at 4 °C.

### 2.3. Preparation of RBP-NaCas and RBPNs-NaCas Composite Protein

The NaCas, RBP, and RBPN stock solutions were prepared with deionized water at a protein concentration of 0.5% (*w*/*v*). Based on the method of Wang et al. [16], the RBP and RBPNs were mixed with the NaCas solution in a ratio of 1:1 (*v*/*v*) and stirred until homogeneous. The pH of the composite protein solutions was adjusted to 7.0. Consequently, two kinds of composite protein systems were formed and named RBP-NaCas and RBPNs-NaCas, respectively.

### 2.4. Particle Size and ζ-Potential Measurements

The Malvern Zetasizer Nano ZS (Malvern Instruments, Worcestershire, UK) was used to determine the particle size distributions and ζ-potential according to a previous study [20]. In brief, the stock solutions were diluted 5-fold to 1.0 mg/mL with 10 mM PBS and adjusted to pH 7.0 before measurements. The refractive indices of protein and dispersant were set to 1.45 and 1.33, respectively. The ζ-potential was automatically characterized after stabilization for 120 s at 25 ± 1 °C, and each measurement was performed in triplicate.

### 2.5. Apparent Viscosity Analysis

The dynamic apparent viscosity of each protein solution was determined as described by Zhang et al. using a Discovery HR-20 rheometer (TA Instruments, New Castle, DE, USA) [21]. Before installing the fixture, the aluminum parallel plate (60 mm in diameter and 1 mm in gap) was accurately calibrated and used throughout the shear rheological test process. The shear rate gradually increased from 1 to 100 s^−1^ at 10 points per order of magnitude and was measured at 25 °C. The resulting shear viscosity was averaged at each speed, and the flow curves were fitted to the power-law model (Ostwald–deWaele equation) using Equation (1).

(1) $$\eta =K\gamma^{n-1}$$

where η, k, γ, and n represent the apparent viscosity (Pa·S), the consistency coefficient (mPa·s^n^), shear rate (s^−1^), and flow behavior properties, respectively.

### 2.6. Foaming Capacity and Foaming Stability

The foaming properties were evaluated by foaming capacity (FC) and foaming stability (FS) according to previous research with necessary modifications [17,22]. The electric stirrer (Zhanyi-ZY3057, Shanghai, China) was immersed approximately 1 cm below the liquid surface to stir 20 mL of protein solutions continuously for 1 min. The foams produced were immediately transferred to a specially calibrated measuring cylinder, and the foam volume was measured immediately and again after standing for 2 h. The FC and FS of the protein solution samples were calculated using Equations (2) and (3), respectively.

(2) $$\mathrm{FC}(\%)=\frac{V_{1}}{20}\times 100$$

(3) $$\mathrm{FS}(\%)=\frac{V_{2}\;-\;20}{V_{1}\;-\;20}\times 100$$

where *V*_1_ is the foam volume after whipping immediately, and *V*_2_ is the foam volume at 2 h after whipping.

### 2.7. Foam Microstructure

The size distribution and morphology of the air bubbles were assessed by a confocal laser scanning microscope (FV1200, Olympus, Tokyo, Japan) [23]. In brief, the fluorescein sodium salt (0.1 mmol/L) was blended with the prepared sample solutions and stained at room temperature for at least 2 h. After whipping, an appropriate portion of stained foam was gently mounted on a concave glass slide (6 mm in diameter and 1.8 mm in depth) and immediately covered with a cover glass. Foam samples were magnified 10 times at an excitation wavelength of 488 nm to observe and record the images every 5 min within half an hour. The captured fluorescence images were statistically analyzed using Image Pro Plus 6.0 software.

### 2.8. Dynamic Surface Pressure

Based on the method of Zhang et al., the surface pressure (π, mN/m) was recorded as the difference in the surface tension between the ultrapure aqueous solution (σ_0_) and the sample solution (σ) [17]. The change in surface tension was determined by the shape analysis of a pendant drop using the Droplet Shape Analyzer (DSA30, Krüss GmbH, Hamburg, Germany). A droplet of protein solution sample was suspended into a transparent quartz glass container via a syringe, and then the drop shapes were recorded by a CCD digital camera for 3600 s and analyzed using the ADVANCE 5.0 software. The surface pressure of the sample droplet over time was fitted by Equation (4).

(4) $$\pi =2C_{0}KT\sqrt{\frac{Dt}{3.14}}$$

where C_0_ is the concentration of the bulk phase, *K* is the Boltzmann constant (1.38 × 10^−23^ J K^−1^), T is the absolute temperature, D is the diffusion coefficient, and t is the adsorption time. If diffusion controls the adsorption process, the plot of π-t^1/2^ will be linear, at which point the slope is the diffusion rate (*K*_diff_).

### 2.9. Interfacial Rheological Properties

The interfacial behaviors were monitored at different deformation amplitudes (ΔA/A) and frequencies (ω) using the oscillating drop method [22]. The records of surface expansion modulus (*E*), interfacial expansion elasticity (*E*_d_), and interfacial expansion viscosity (*E*_v_) were obtained using the same instruments and software as described in Section 2.8 by compression and expansion of a periodic sinusoidal interface. The samples were diluted to 0.1% (*w*/*v*) with deionized water with the same pH of 7.0, placed in a transparent quartz glass container at 25 °C, and stabilized for 600 s. The amplitude sweep was performed at a fixed frequency of 1 Hz by increasing from 1% to 18%. The frequency sweep was performed ranging from 0.1 to 1 Hz at a constant deformation amplitude of 3%. The variation of the surface dilatational modulus (*E*) over time was monitored between 0 and 1800 s at a fixed frequency of 1 Hz and amplitude of 3%. The surface dilatation modulus (*E*) was determined by Equation (5). Numerically, the absolute value of the modulus of expansion (|*E*|) can be calculated from Equation (6).

(5) $$E=\frac{d\sigma }{dA/A}=-\frac{d\pi }{d\ln A}=E_{d}+iE_{v}$$

(6) $$\left|E\right|=\sqrt{{E_{d}}^{2}+{E_{v}}^{2}}$$

### 2.10. Statistical Analysis

All experiments were conducted at least three times, unless otherwise noted. The data were expressed as mean ± standard deviation (SD) and plotted using Origin 2018 software (OriginLab, Northampton, MA, USA). Statistical analyses were performed with SPSS software (SPSS 19.0, IBM, Chicago, IL, USA), and the considered statistically significant level was *p* < 0.05.

## 3. Results and Discussion

### 3.1. Particle Size and the ζ-Potential

**Figure 1:**
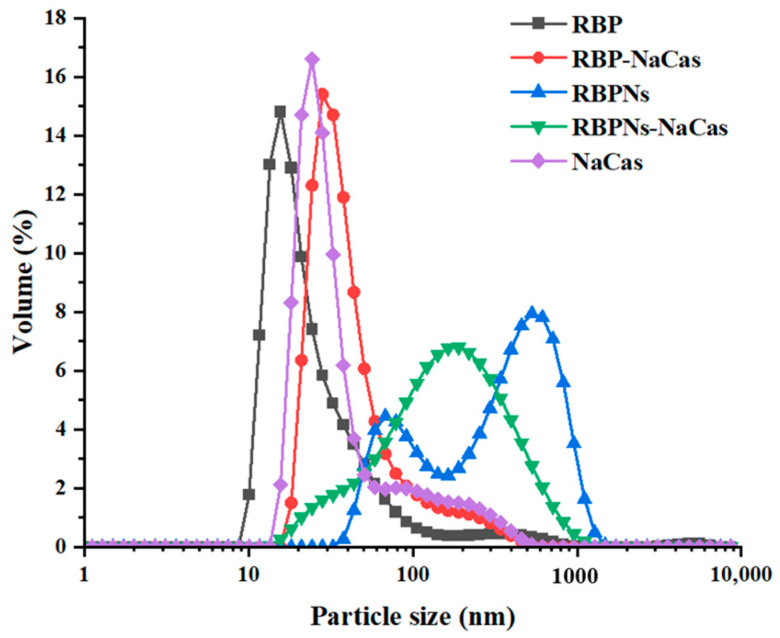
Particle size distribution. RBP: native rice bran protein; RBPNs: RBP nanoparticles; NaCas: sodium caseinate; RBP-NaCas: composite protein system composed of RBP and NaCas; RBPNs-NaCas: composite protein system composed of RBPNs and NaCas.

The particle size distribution of protein samples is shown in Figure 1, in which the volume peaks of RBP, NaCas, and RBP-NaCas composite protein were mainly single peak and concentrated at 27.6 nm, 30.1 nm, and 55.7 nm, respectively, and the RBP-NaCas had similar peaks width and intensity to those of individual proteins, suggesting the existence of interaction between RBP and NaCas in the RBP-NaCas composite protein. However, when RBPNs and NaCas were mixed, the particle size of RBPNs-NaCas showed a broader single-peak particle size distribution that was close to the second peak of RBPNs, thus indicating that the presence of NaCas weakened the aggregation of RBPNs in aqueous solution. Another study also found that the larger aggregates of WPI-NaCas could be significantly reduced after complexation [24].

The ζ-potential is a commonly used term to describe the electrostatic interaction between charged particles. As shown in Table 1, the absolute ζ-potential values of RBP and RBPNs were lower than those of NaCas. After mixing NaCas with RBP and RBPNs, the ζ-potential values of the composite protein solution were compared to those of the NaCas, RBP, and RBPNs solutions, which further confirmed the protein–protein interaction in the composite protein solution.

The results of both particle size distribution and ζ-potential showed that the two kinds of rice bran protein could interact with NaCas after mixing at pH 7.0, which is mainly due to the fact that protein and protein particles have both positive and negative charged patches [18,24]. The interaction of RBP and RBPNs with NaCas through the opposite surface charge could change the physicochemical properties of the composite protein system, which affected the rheological properties, interfacial properties, and foaming properties.

### 3.2. Apparent Viscosity

**Figure 2:**
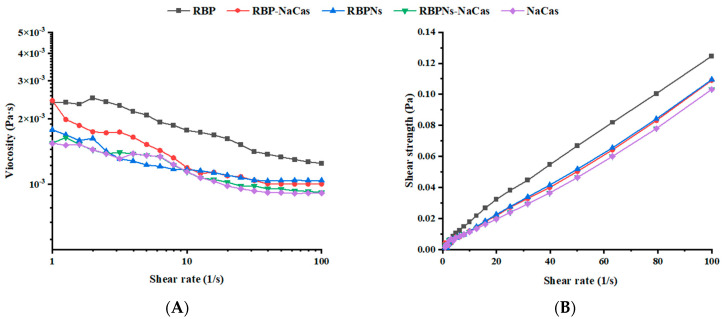
(**A**) Shear viscosity and (**B**) shear strength as a function of shear rate. RBP: native rice bran protein; RBPNs: RBP-nanopartic les; NaCas: sodium caseinate; RBP-NaCas: composite protein system composed of RBP and NaCas; RBPNs-NaCas: composite protein system composed of RBPNs and NaCas.

**Figure 3:**
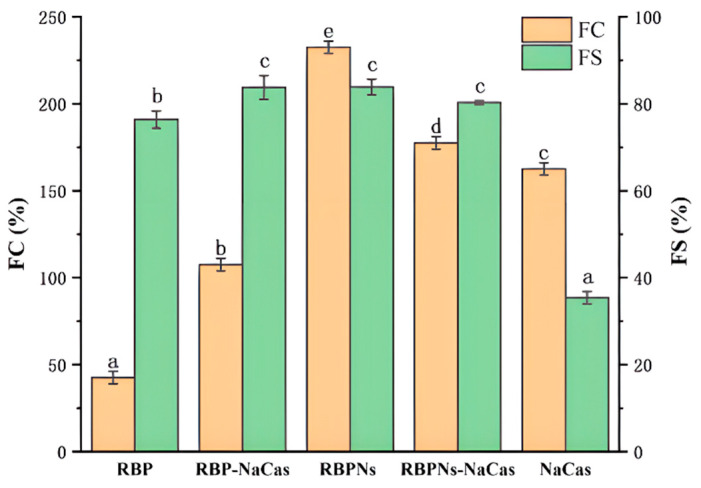
The foaming capacity (FC) and foaming stability (FS). Different letters indicate significant differences (*p* < 0.05). RBP: native rice bran protein; RBPNs: RBP nanoparticles; NaCas: sodium caseinate; RBP-NaCas: composite protein system composed of RBP and NaCas; RBPNs-NaCas: composite protein system composed of RBPNs and NaCas.

As the shear rate scanning increased from 1 to 100 s^−1^, the apparent viscosity exhibited a significant decreasing trend (see Figure 2A), and the shear strength increased almost linearly (Figure 2B), showing a typical shear-thinning behavior. The parameters obtained by fitting the shear viscosity curve with the power-law model are presented in Table 2. The consistency coefficient (*K*) represents the flow resistance, and the fluidity index (n) can reflect the fluidity of the solution. The n values of the protein solutions were all less than 1, indicating that they all belonged to pseudoplastic fluids in power-law fluids. The *K* value and n value of RBP-NaCas were intermediate between RBP and NaCas, which was basically in accordance with the foaming properties of these three proteins (Figure 3). Compared to RBP, RBP-NaCas exhibited lower apparent viscosity and *K* value, indicating that the RBP-NaCas with low flow resistance resulted in easier disruption with increasing shear rate, which is beneficial for the incorporation of bubbles and molecular diffusion rate, thereby improving the FC and adsorption efficiency of the RBP-NaCas. Therefore, NaCas possessed with flexible structure may play a role similar to “chaperone activity” in composite protein systems, assisting in recombination of protein conformation and altering the interactions of proteins [24]. The apparent viscosity has a significant influence on the fluidity of the continuous phase surrounding the bubble, which is closely related to the liquid drainage and coalescence of the foam system, thus affecting the stability of the foam [25]. However, the FS of RBP-NaCas was significantly better than that of RBP and NaCas (*p* < 0.05). More interestingly, the RBPNs-NaCas with better foaming properties showed similar parameters of the power-law model and apparent viscosity as NaCas (Figure 2 and Table 2). It could be inferred that the foaming properties of composite proteins may be dominated by their interfacial properties.

### 3.3. Analysis of the Foaming Properties

The foaming capacity (FC) and foaming stability (FS) of RBP, RBPNs, NaCas, and composite proteins are determined in Figure 3. The lowest FC was found for the individual RBP, merely at 42.5%, while the FS was relatively high, which was consistent with the previous research [17]. The FS of RBP-NaCas was 48.4% higher than that of NaCas, and there was a 65% increase in FC compared to RBP. It is reported that the prerequisite for optimal foaming properties is the rapid formation of a cohesive air–water interfacial film [26,27]. The noticeable increase in resistance of the RBP-NaCas system to bubble drainage and collapse may be due to higher surface activity and the formation of a thickened adsorption layer. Some studies have shown that the complex systems of NaCas, such as tilapia protein isolate–NaCas [28], typically exhibited excellent FS after complexation, but the FC was extremely limited, even drastically reduced. In comparison, after the complexation of RBPNs and NaCas, the FC and FS of NaCas significantly increased from 162.5% and 35.4% to 177.5% and 80.3% (*p* < 0.05), which demonstrated impressive improvement of foaming properties. Cao et al. found that rigid spherical proteins can form a more viscoelastic interfacial film than flexible random helical proteins, while flexible proteins have faster adsorption rates, which is essential for improving the FC [29]. The same research group in this study reported that the conformation of RBPNs can be unfolded after thermal acid treatment [18], and other studies have also discovered that NaCas has a favorable flexible structure [20]. Therefore, it could be speculated that the unfolding and the conformational adjustment of flexible structure made it easier for RBPNs-NaCas to adsorb and interact at the interface, resulting in better FC. In addition, the low net charge of RBPNs-NaCas (Table 1) weakened the intermolecular mutual repulsion and facilitated the protein interaction at the air–water interface. Therefore, the improvement in FS of RBPNs-NaCas could be attributed to the formation of a multilayer protein molecular network structure and reinforcement of the viscoelastic properties of the interfacial films. These results again indicate that the formation of viscoelastic interfacial film may be the main reason for the improvement of the foaming properties of composite proteins, and it will be discussed in Section 3.6 of this study.

### 3.4. Foam Microstructure

**Figure 4:**
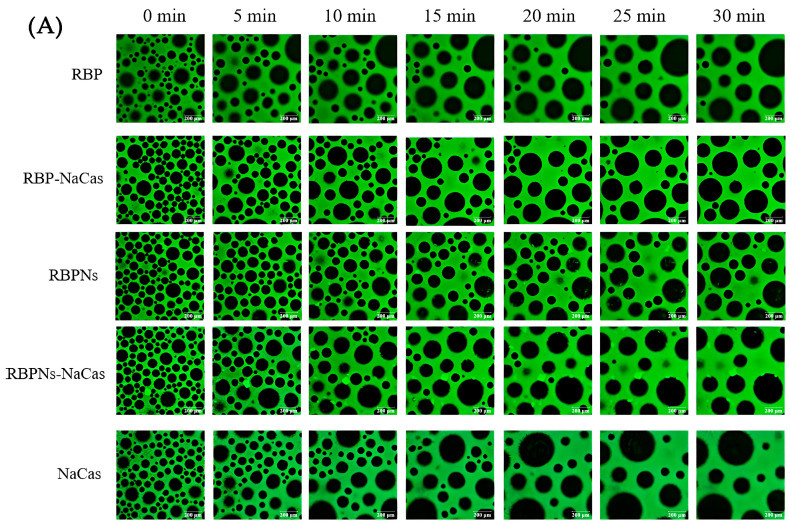

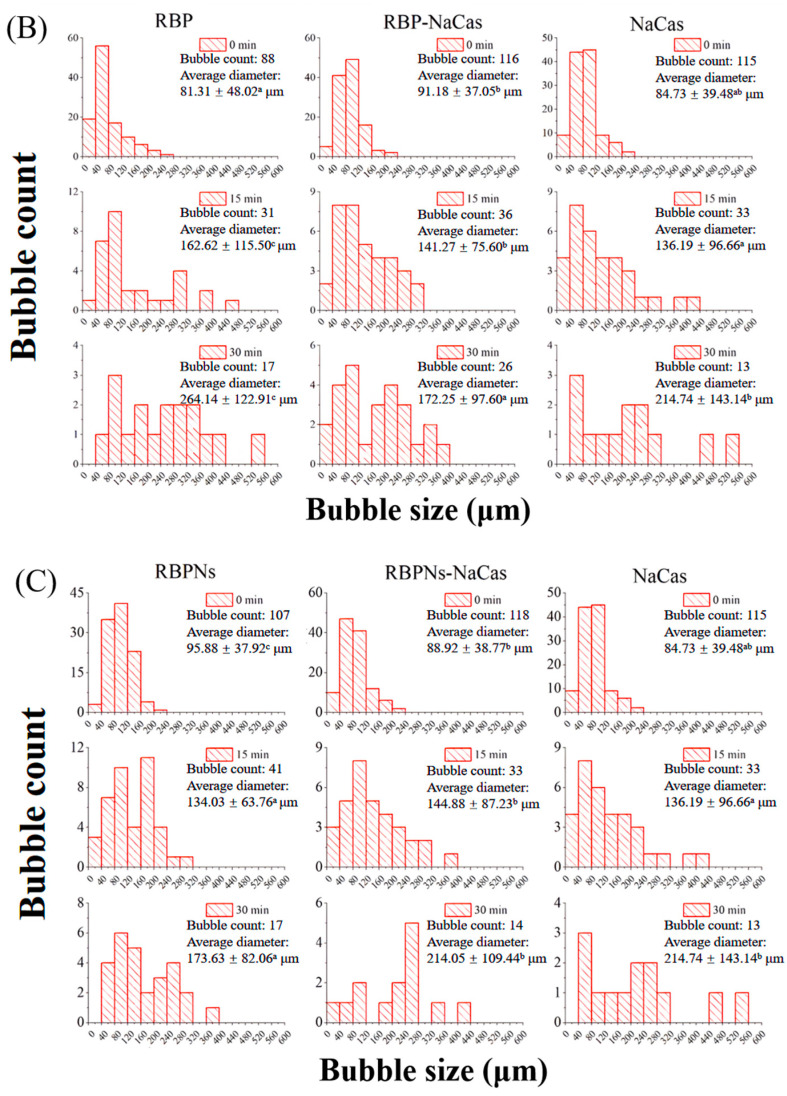
(**A**) Microstructure evolution of protein foams. Green is protein, and black is air bubble. The scale is set to 200 μm. (**B**) Histograms of bubble diameter and bubble count of RBP, NaCas, and RBP-NaCas as a function of time (t). (**C**) Histograms of bubble diameter and bubble count of RBPNs, NaCas, and RBPNs-NaCas as a function of time (t). Different letters denoted as superscripts indicate significant differences (*p* < 0.05). RBP: native rice bran protein; RBPNs: RBP nanoparticles; NaCas: sodium caseinate; RBP-NaCas: composite protein system composed of RBP and NaCas; RBPNs-NaCas: composite protein system composed of RBPNs and NaCas.

The stable interfacial structure of bubbles is related to their count and size distribution; meanwhile, the destabilization process of the foam can be investigated by the microstructure to further evaluate the FC and FS of the protein samples. As shown in Figure 4A, the bubble count of freshly prepared RBP was relatively low and formed a dispersed or disordered arrangement structure, which was consistent with its poor FC (Figure 3). In contrast, the RBPNs, RBP-NaCas, and RBPNs-NaCas solutions formed more bubbles. With the extension of time, the bubble count of all samples decreased, and the diameter increased to varying degrees, showing a typical phenomenon known as foam coarsening. After 30 min of storage, the foam coarsening rates of RBP-NaCas and RBPNs-NaCas stabilized foam were significantly slower than those of NaCas, indicating that the composite proteins may have formed a more compact air–liquid interface layer, which was conducive to the long-term FS.

The variation in bubble size distribution reflects the foaming properties to a large extent, as liquid drainage rates and unstable foam systems are usually characterized by larger bubbles. The diameter distribution histogram (Figure 4B,C) shows that, within half an hour, the average foam diameters of RBP, RBPNs, NaCas, RBP-NaCas, and RBPNs-NaCas increased by 202.7%, 81.1%, 153.4%, 88.9%, and 140.7%, respectively. During the initial foaming stage, the bubble count of RBP-NaCas was higher than that of RBP and NaCas, while maintaining more foam volume and a narrower size distribution range after 30 min. The evolution of these foam microstructures showed that the complexation of RBP and NaCas promoted the interaction between the air–water interface, thereby slowing down the destabilization process of the foam. This may be due to the interfacial film of the RBP-NaCas providing an energy barrier to prevent disproportionation and condensation, so that bubbles still showed a certain degree of stability. Although the parameters of average bubble diameter and bubble count of RBPNs-NaCas were inferior to those of RBPNs, there was no significant difference in the FS between RBPNs and RBPNs-NaCas (*p* < 0.05), indicating that the interaction of the two proteins slowed down the coarsening and flocculation phenomena in the foam system. According to the study of Langevin D., coalescence refers to the rupture of membranes between bubbles or emulsions [4]. Compared to the NaCas foam system, RBPNs-NaCas had slightly more bubbles and a concentrated diameter distribution. The RBPNs may have endowed NaCas with better interface viscoelastic behaviors, resulting in lower coalescence and Ostwald ripening of RBPNs-NaCas foams. However, coalescence occurs rapidly and is associated with other phenomena, such as foam drainage caused by gravity, Ostwald ripening, etc. It is also hypothesized that the larger particle size of RBPNs-NaCas may have hindered the foam drainage by blocking the plateau channels, so that the foam system showed reliable stability.

### 3.5. Adsorption Kinetics

**Figure 5:**
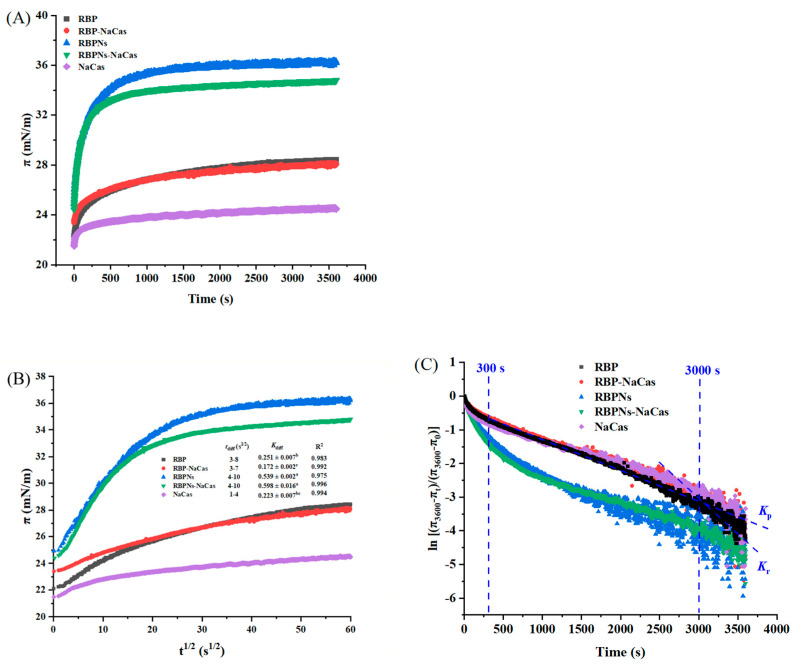
Characteristic diagram of adsorption kinetics. (**A**) Surface pressure (π) as a function of time (t). (**B**) Plots of surface pressure (π) vs. square root of time (t^1/2^), where *t*_diff_ represents diffusion time and *K*_diff_ means diffusion rate. (**C**) [(π_3600_-π_t_)/(π_3600_-π_0_)] of as a function of time (s), the slope of the first (*K*_p_) and the second (*K*_r_) linear regions indicates molecular permeation (300–3000 s) and structural rearrangement (3000–3600 s) of protein samples. Different letters denoted as superscripts indicate significant differences (*p* < 0.05). RBP: native rice bran protein; RBPNs: RBP nanoparticles; NaCas: sodium caseinate; RBP-NaCas: composite protein system composed of RBP and NaCas; RBPNs-NaCas: composite protein system composed of RBPNs and NaCas.

The properties of foams are typically related to the interfacial adsorption kinetics and the rheological properties of the interface [20]. Figure 5A shows the surface pressure (π) as a function of time, which can be roughly divided into three stages, i.e., absorption induction (0–25 s), rapid diffusion (25–600 s), and gradual equilibration (after 600 s). Related studies have shown that a higher equilibrium π value indicates a better FC [25,30]. After reaching equilibrium, the π values of RBP, RBP-NaCas, RBPNs, RBPNs-NaCas, and NaCas are 28.4 mN/m, 28.1 mN/m, 36.2 mN/m, 34.8 mN/m, and 24.5 mN/m, respectively, while the FC of NaCas was not as low as expected (Figure 3). Moreover, there was no significant difference in FC between RBPNs-NaCas with lower equilibrium π values and RBPNs (*p* < 0.05), and a similar situation was also observed in a study of sodium caseinate and whey protein concentrates (NaCas-WPC) [31]. These phenomena may be related to the processes of protein adsorption and rearrangement at the interface. In this situation, the Ward and Tordai model was used to elucidate the diffusion kinetics of surfactants [32]. In the protein composite system with low concentration, the adsorption kinetics at the early stages are governed by the diffusion process driven by the concentration gradient. Therefore, the migration of protein particles at the air–water interface can be reflected by the diffusion rate constant (*K*_diff_) in the linear part of the π-t^1/2^ curve [25]. Theoretically, a higher *K*_diff_ rate is associated with higher protein surface activity. The diffusion rate of NaCas increased dramatically from 0.223 ± 0.007 to 0.598 ± 0.016 after complexing with RBPNs, and these results were reasonable because Wang et al. also found that the diffusion of Cas-SPNP at the interface was more dependent on the flexible molecules of NaCas unfolding on the interfacial film [16]. In addition, there were some non-protein components (such as phenols) in RBP that could be involved in competitive protein adsorption or interact with proteins at the interface, thereby affecting the surface properties of RBP-NaCas, which gave RBPNs-NaCas a more pronounced surface activity advantage in the diffusion phase, resulting in a more stable adsorption equilibrium [33].

With the time of adsorption, the initial adsorption behavior of the protein solution was not completely controlled by the protein diffusion rate, and the π-t^1/2^ curve gradually deviated from the linear state; thereby, the rates of permeation (*K*_p_) and rearrangement were used to analyze the adsorption kinetics at the long-term water–liquid interfaces of protein molecules. Related studies have found that FC is closely related to the *K*_p_ of proteins, and the *K*_r_ of active molecules at the interface can also significantly affect FS [34]. As shown in Figure 5C and Table 3, the *K*_r_ of all samples was significantly higher than *K*_p_, indicating that the rearrangement process dominated the dynamic adsorption behavior of the composite proteins. The NaCas with the highest *K*_r_ absolute value showed a good FC. This may be due to the flexible structure of NaCas, which facilitated faster adsorption to the interface and better FC [16]. Moreover, the repulsive interaction of the NaCas molecule due to the higher net charge slowed down the *K*_diff_ (Figure 5B), and the rearrangement rapidly occurred before the formation of a compact and viscoelastic interfacial film (Figure 5C and Table 3), leading to the insufficient FS. It is reported that the phase separation of protein biopolymers can lead to faster interfacial penetration [35]. Therefore, the *K*_p_ values of RBPNs-NaCas and RBP-NaCas with a higher degree of polymerization were much higher than those of NaCas, which also favored the foaming ability of the complexes. After the RBP-NaCas penetrated the interface, the *K*_r_ absolute value increased dramatically. Feng et al. also prepared the zein/NaCas nanocomplexes and found that the NaCas can be competitively adsorbed to the interface, and the hybrid interfacial structure was rearranged [36]. Therefore, the flexible structure of NaCas could have induced a higher degree of unfolding and interaction of composite proteins at the interface, which was conducive to the formation of a more elastic interfacial film, thus improving the FS of the NaCas-based composite system.

### 3.6. Dilatational Rheology Properties

#### 3.6.1. Amplitude Sweep

**Figure 6:**
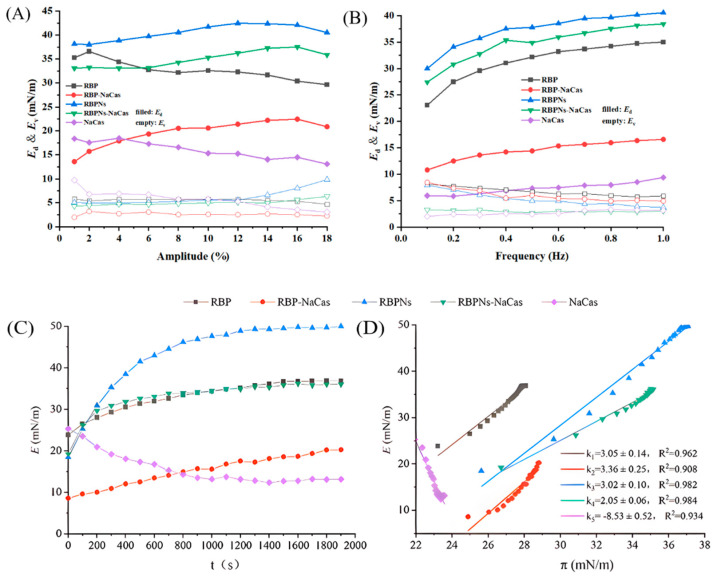
Changes in surface elasticity modulus (*E*_d_) and surface viscosity modulus (*E*_v_) with different amplitudes (**A**) and frequencies (**B**). Changes in surface dilatational modulus (*E*) with time (**C**) and surface pressure (**D**). RBP: native rice bran protein; RBPNs: RBP nanoparticles; NaCas: sodium caseinate; RBP-NaCas: composite protein system composed of RBP and NaCas; RBPNs-NaCas: composite protein system composed of RBPNs and NaCas.

The measurement of surface dilatational modulus provides information about the non-equilibrium states of the surface, the structural states of adsorbed proteins, molecular interactions, etc. To further analyze the interfacial properties of RBP-NaCas and RBPNs-NaCas, an amplitude sweep was performed. Figure 6A reveals that the *E*_d_ values for each protein sample were markedly higher than those of *E*_v_, which demonstrated that the *E* values were mainly contributed by *E*_d_ values and the adsorption layer formed at the air–water interface behaved mainly as an elastic film within the tested amplitude range. Therefore, it would like to focus on the analysis of the amplitude dependence of the *E*_d_. Within the deformation amplitude range of 1% to 18%, the *E*_d_ values of RBP-NaCas and RBPNs-NaCas showed a gradually increasing trend with amplitude, indicating that the nonspecific interaction between interfacial proteins may facilitate the formation of a liquid film with better tensile properties and rigidity at the interface, which was closely related to the better FS. Comparing with NaCas-stabilized interfaces, the RBPNs-NaCas had a higher *E*_d_ value, increasing from 33.08 to 37.52 mN/m, suggesting that the enhanced interaction between interfacial proteins promoted the formation of a more viscoelastic network structure. In fact, the addition of RBP changed the downward trend of the viscoelasticity of NaCas, and the elasticity of RBP-NaCas (19.35 to 20.88 mN/m) was significantly higher than that of NaCas when the amplitude exceeded 4% as well. Researchers found that some small molecules can act as “bridging agents” to improve the interaction between protein molecules [37]. It is hypothesized that RBP plays a similar role in the composite protein system, which strengthens the network connectivity of RBP-NaCas at the interface, resulting in the formation of a more elastic interfacial adsorption film at the interface. It is also worth noting that when the amplitude exceeded 16%, the *E*_d_ value of all samples slightly decreased, suggesting that higher amplitude deformation can significantly disrupt the structure of the interfacial film, leading to structural rearrangement during sinusoidal periodic oscillation [38] and an increase in foam drainage rate.

#### 3.6.2. Frequency Sweep

As shown in Figure 6B, the result of the frequency sweep was consistent with the amplitude sweep, and the surface dilatational modulus was also dominated by the *E*_d_ value and increased with frequency, further confirming the typical elastic response and frequency dependence behavior within the interfacial films of RBP-NaCas and RBPNs-NaCas. Correlation studies demonstrate that frequency dependence is an essential characterization of non-covalent gels [39]. Therefore, the non-covalent hydrophobic and electrostatic interactions may play an important role in the composition of RBP-NaCas and RBPNs-NaCas. This interfacial behavior was mainly caused by the relaxation phenomenon and structural changes in the interfacial film. On the one hand, the relaxation process in the film was more pronounced due to the ample time that composite protein molecules had to rearrange or exchange at relatively low frequencies, resulting in higher viscosity behavior [40]. On the other hand, the high frequency caused a shortened response time for the interfacial deformation, resulting in a rapid rearrangement of the molecular structure at the interface and a higher elastic modulus of the interfacial layer [41]. In addition, the interfacial layer stabilized by RBPNs-NaCas had a much higher *E*_d_ value (27.41 to 38.41 mN/m) compared to NaCas (5.85 to 9.38 mN/m) at frequencies ranging from 0.1 to 1.0 Hz, showing that the addition of RBPNs increased the rapid rearrangement of NaCas and its resistance to external deformation. Similar trends were observed in the surface dilatational modulus changes of the RBP-NaCas system, suggesting that the complex of RBP and NaCas also enhanced the intermolecular interactions and facilitated the migration of RBP-NaCas toward the interface to change the rigidity of the film layer at the interface. From the above analysis, it can be concluded that the RBP-NaCas and RBPNs-NaCas were capable of forming a more viscoelastic interfacial adsorption layer in response to external deformation, which improved the FS of the NaCas system.

#### 3.6.3. The Effect of Interactive Force on Interfacial Behavior

The surface dilatational modulus as a function of time at a fixed amplitude and frequency (3%, 1 Hz) was investigated. As shown in Figure 6C, the *E* value of NaCas gradually decreased with time to an equilibrium value of approximately 13.15 mN/m, indicating that the adsorption layer of NaCas was susceptible to sinusoidal periodic oscillatory deformations and disruption of the interfacial structure, while the *E* curves of RBP-NaCas and RBPNs-NaCas exhibited an increasing trend until approaching an equilibrium value at 36.16 mN/m and 35.28 mN/m, respectively, indicating that the intermolecular interactions between molecules increase with the adsorption of composite proteins, and the interfacial behaviors of composite proteins were dominated by RBP and RBPNs components. The study of the alcohol-free zein-sodium caseinate composite found that the structure of the interfacial viscoelasticity network can be formed rapidly after complexation [15]. The RBP and RBPNs, as surfactants, also played similar roles, and the network rearrangement of adsorbed composite protein molecules at the interface contributes to an increase in density and elasticity of the interfacial film, all of which prevented the expansion, shrinkage, movement, or coalescence of NaCas bubbles [17].

The *E*-π curve and its slope can provide information on the intermolecular interactions of protein samples and the adsorption load within the film of protein particles [42]. As described in Figure 6D, the data points are concentrated at higher interfacial pressure, indicating that the diffusion-controlled migration at the early stage was much faster than the later permeation and rearrangement. In general, the ideal equilibrium adsorption state of the slope derived from the *E*-π curve should be around 1 [43]. The *E*-π curve slope of NaCas was less than 1, indicating that the adsorption of NaCas molecules at the interface was in a non-ideal state and the intermolecular interaction was weak. Therefore, the mechanical strength of the interfacial film formed by NaCas was insufficient, resulting in poor FS. The results showed the following two aspects: On the one hand, the dependence between the *E* and π values of the composite protein demonstrated an increase in protein adsorption on the liquid film, resulting in a denser adsorption layer. On the other hand, in contrast to the negative value of the *E*-π curve slope of NaCas, the slope of RBP-NaCas and RBPNs-NaCas was much greater than 1, indicating that the adsorption at the interface was in a non-ideal state. The existence and increase of high protein–protein interaction in the composite proteins significantly affected the molecular structure condensation of NaCas, RBP, and RBPNs adsorbed at the interface. Therefore, these observed phenomena were consistent with the trend of FS changes.

#### 3.6.4. Interface Behavior Mechanism of Composite Protein Systems

Based on the above analysis about interfacial properties, the interface behavior mechanisms by which RBP and RBPN particles improve the foaming properties of NaCas systems were mainly categorized into the following: (1) RBP, RBPNs, and NaCas particles were directly adsorbed on the air–water interface, resulting in a particle-covered foam interfacial surface layer, which served to protect individual bubbles from unfavorable factors such as coalescence, Ostwald ripenin, etc. (2) After the adsorption of RBP-NaCas and RBPNs-NaCas complex protein particles at the interface, the complex particles formed an interfacial membrane at the interface through the bridging effect of sodium caseinate. In addition, the complex particles in the continuous phase can hinder foam drainage by blocking the plateau channels. The mechanism of interfacial membrane formation of the complex protein is shown in Figure S1.

## 4. Conclusions

To sum up, the RBP and RBPNs can serve as potential natural surfactants to facilitate the foaming and interfacial properties of NaCas. The size distribution, ζ-potential, and shear consistency coefficient results proved that the interfacial adsorption behavior of RBP-NaCas and RBPNs-NaCas was significantly improved due to the strong electrostatic intermolecular interactions. The evolution of the foam microstructure also demonstrated that RBP and RBPNs could effectively stabilize the bubble size of NaCas and retard foam drainage and coarsening. The flexible structure of NaCas enabled the composite protein molecules to rapidly permeate and rearrange at the air-water interface, which enhanced the FC of RBP-NaCas. In addition, the RBP-NaCas and RBPN-NaCas composites formed a more viscoelastic composite film at the air–water interface than the monolayer NaCas, which significantly improved the foaming properties of NaCas. These results provide a theoretical foundation for the processing and application of the RBPNs-NaCas and RBP-NaCas foam systems, so as to explore the potential utilization of natural and healthy foaming agents in foamed food manufacturing.

## Figures and Tables

**Table 1:** ζ-potentials of RBP, RBPNs, NaCas, and composite proteins.

Protein Samples	Zeta Electric Potential (mV)
RBP	−31.4 ± 0.15 ^b^
RBP-NaCas	−35.5 ± 0.07 ^a^
RBPNs	−22.3 ± 0.26 ^d^
RBPNs-NaCas	−27.2 ± 0.28 ^c^
NaCas	−36.8 ± 1.06 ^a^

Note: Different letters denoted as superscripts indicate significant differences (*p* < 0.05). RBP: native rice bran protein; RBPNs: RBP nanoparticles; NaCas: sodium caseinate; RBP-NaCas: composite protein system composed of RBP and NaCas; RBPNs-NaCas: composite protein system composed of RBPNs and NaCas.

**Table 2:** Parameters from the power-law model. The consistency coefficient (*K*) represents the flow resistance, and the fluidity index (n) can reflect the fluidity of the solution.

Protein Samples	*K* (×10^3^)	n	R^2^
RBP	3.05 ± 0.28 ^c^	0.817 ± 0.010 ^a^	0.993
RBP-NaCas	2.09 ± 0.18 ^b^	0.806 ± 0.012 ^a^	0.963
RBPNs	1.68 ± 0.11 ^a^	0.866 ± 0.015 ^b^	0.985
RBPNs-NaCas	1.62 ± 0.25 ^a^	0.856 ± 0.012 ^b^	0.949
NaCas	1.60 ± 0.12 ^a^	0.853 ± 0.012 ^b^	0.937

Note: Different letters denoted as superscripts indicate significant differences (*p* < 0.05). RBP: native rice bran protein; RBPNs: RBP nanoparticles; NaCas: sodium caseinate; RBP-NaCas: composite protein system composed of RBP and NaCas; RBPNs-NaCas: composite protein system composed of RBPNs and NaCas.

**Table 3:** Characteristic dynamic parameters of adsorption at the interfacial layer, including penetration rate *(K*_p_), structural rearrangement (*K*_r_) at the interface, and the initial and final interfacial pressure (π_0_ and π_3600_).

Sample	*K*_p_ (×10^4^)	*K*_r_ (×10^4^)	π_0_	π_3600_
	(s^−1^)	(s^−1^)	(mN/m)	(mN/m)
RBP	−8.159 ± 0.014 ^cd^	−13.210 ± 0.357 ^c^	22.11± 0.22 ^c^	28.50 ± 0.23 ^b^
RBP-NaCas	−8.331 ± 0.017 ^c^	−18.402 ± 0.651 ^a^	23.42 ± 0.15 ^b^	28.16 ± 0.34 ^b^
RBPNs	−10.501 ± 0.063 ^a^	−14.243 ± 1.102 ^b^	24.94 ± 0.12 ^a^	36.39 ± 0.27 ^a^
RBPNs-NaCas	−9.606 ± 0.050 ^b^	−14.100 ± 0.289 ^bc^	24.41 ± 0.26 ^ab^	34.85 ± 0.15 ^a^
NaCas	−7.259 ± 0.018 ^d^	−18.501 ± 0.567 ^a^	21.49 ± 0.16 ^c^	24.59 ± 0.18 ^c^

Note: Different letters denoted as superscripts indicate significant differences (*p* < 0.05). RBP: native rice bran protein; RBPNs: RBP nanoparticles; NaCas: sodium caseinate; RBP-NaCas: composite protein system composed of RBP and NaCas; RBPNs-NaCas: composite protein system composed of RBPNs and NaCas.

## Data Availability

The data presented in this study are available on request from the corresponding author. The original data in this article are not publicly available due to privacy reasons.

## Supplementary Materials

The following supporting information can be downloaded at: https://www.mdpi.com/article/10.3390/foods13152328/s1. Figure S1: Schematic illustration of the adsorption mechanism of the foams whipped by RBP-NaCas and RBPNs-NaCas at the air–water interface. RBP-NaCas: composite protein system composed of rice bran protein and sodium caseinate; RBPNs-NaCas: composite protein system composed of RBP nanoparticles and NaCas.
