# Supplementary material for: Effect of Rice Bran Protein on the Foaming Properties and Foaming Characteristics of Rice Bran Protein–Sodium Caseinate and Rice Bran Protein Nanoparticles–Sodium Caseinate
Source: Foods. 2024 Jul 24;13(15):2328. doi: 10.3390/foods13152328 (PMC11311429; doi:10.3390/foods13152328)
Supplement: Supplementary file 1 [file foods-13-02328-s001.zip › foods-3077399-supplementary.pdf]

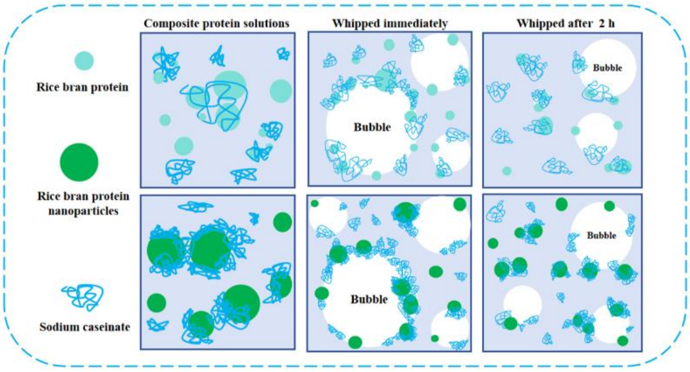

Figure S1. Schematic illustration of the adsorption mechanism of the foams whipped by RBP-NaCas and RBPNs-NaCas at the air-water interface. RBP-NaCas: composite protein system composed of rice bran protein and sodium caseinate; RBPNs-NaCas: composite protein system composed of RBP-nanoparticles and NaCas.
